# Rural tourism networking and covid-19 crisis: a gender perspective

**DOI:** 10.1007/s11628-022-00503-x

**Published:** 2022-08-27

**Authors:** Ana M. Morales-Hernández, Carlos Fernández-Hernández, Flora M. Díaz-Pérez, Carlos G. García-González

**Affiliations:** 1 Facultad de Ciencias Jurídicas, Universidad de Las Palmas de Gran Canaria, Las Palmas de Gran Canaria, Spain; 2 Facultad de Economía, Empresa y Turismo, Universidad de La Laguna, 38071 La Laguna, Tenerife Spain

**Keywords:** Networking, Rural tourism, Gender, Covid-19 crisis, Island of La Palma

## Abstract

This paper studies networking in rural tourism from a gender perspective comparing pre and post Covid period. The empirical study consisted of a questionnaire answered by 154 tourism promoters. Results showed a higher percentage of promoters belonging to an association during the Covid-19. There was a link between receiving training and belonging to an association and a significant relationship between participation in associations and receiving public funds, this relationship being found more among male promoters. On the other hand, a higher score was given by women for the advantages of networking, and mainly for the following: ‘Simplifies the management of booking’.

**Supplementary Information:**

The online version contains supplementary material available at 10.1007/s11628-022-00503-x.

## Introduction

The development of rural areas is built upon intangible and constantly shifting factors that, through social relations formed in a specific territory, foster a local dialectic. This, in turn, produces a specific type of social capital and a local self-identity (Lee et al. 2005). Local networks articulate the information flow, resources, and identities for the production of beneficial rural development (Jesus and Franco 2016).

On the other hand, the study of rural development and the role that social networks play in such development is particularly relevant in periods of crisis (Schweitzer et al. 2009). In the current century, two economic crises have had an impact on the rural world. First, there was the economic crisis of 2008 and more recently, the health crisis due to Covid-19, which has also led to a major economic crisis (Carli 2020; Mahajan et al. 2020; Kyrgidou 2021; Untarua and Hanb 2021; Carracedo et al. 2021; Coros et al. 2021). As these two crises are so different, the analysis of their respective effects on the activity of tourism promoters in the rural world is of relevance, in order to appreciate the different behaviours in each case. At the same time, given the parameters of the functioning of the rural world, it is essential to observe the behavior of the agents in terms of gender. Indeed, gender is a variable with a crucial role in explaining rural development (Manolova et al. 2020; Phillipson et al. 2020).

Additionally and according to Latour’s actor-network theory (Latour 2007), in order to properly understand the network of social relationships, it is not only the influences among the different actors and their contexts that we have to determine, but the tangible and intangible elements that act as a medium for actors and orient their interests, actions, and connections as well. The interactions between different nodes within a network are based on the actors’ position of power, their resources and knowledge, their perception of the matter in question and other participants who partake in it, and the rules of the interaction itself, among other aspects characterized by each actor’s role (Caalders, 2002; Hwang and Stewart 2017).

In the current globalized world, association appears as a strategy for individuals and local businesses to develop (Luis Busson 2012). Some elements that influence the formation of networks are the participation of individuals in different types of associations, which allows them to acquire a broader list of contacts and the subsequent access to benefits such as resources and information, as well as being able to participate in the decision-making in the fields they are concerned with (Verbole 2003).

Among the associations present in rural environments, we may find rural tourism associations, whose objective is to boost and develop this economic activity through promoters’ participation. Doing so sets the path for networks born from this activity to institutionalize and thus constitute formal networks for families and individual promoters to resort to, if their local informal networks become unable to help them develop their activity further (Francès 2007).

The atomization of rural tourism enterprises, their small dimension, and their low level of promoters’ professionalization are features that condition the supply of rural tourism, and subsequently limit intermediate agencies’ interest in their commercialization (Francès 2006). Due to this issue, new associative models have appeared among promoters, which have been more or less in accordance with the public administration's management, promotion, and marketing policies for the sector. Some of the reasons behind the formation of these entities are the integration of several actors into a single image and action strategy, the possibility of joint promotional and commercial acts, the creation of a centralized reservation system, access to training, becoming eligible for grants and subsidies, the implantation of higher-quality systems, receiving technical support and assessment, and so on (Cassel and Pettersson 2015).

Belonging to a rural tourism association also aids in maintaining a cooperative attitude, so that the resulting human resources generate advantages that can assure the economic viability of the services they offer. Both the cost center (for provisions, input, and services, among others) and the profit center (reserves, activities, integrated packages) represent tangible economic advantages, among other scale economies, that could not be achieved otherwise.

On another matter, there are certain sociodemographic features that condition a person’s participation in associations and, among them, one of the most important is gender. In this regard, it appears to be that men and women approach networking differently depending on the objectives of the association. Therefore, women tend to have a higher presence in non-governmental organizations, parent associations, and religious ones. These types of associations have a public dimension but do not hold a significant amount of political or economic power and simultaneously conform to the traditionally female-dominated aspects of life. On the other hand, men generally participate more in political parties, unions, sports associations, and scientific and professional organizations (Figueroa-Domecq et al. 2020).

From another point of view, it has been shown that men and women are not represented equally when participating in associations, resulting in unequal access to the benefits of this social capital. In this field, women’s public representation is traditionally lower than that of men, which have normally been the visible focus of public spaces. Nevertheless, women’s participation in rural associative structures remains high (Lee et al. 2005).

Moreover, the instruction of human capital that participates in the development of rural tourism is crucial (Zhao et al. 2011; Kyrgidou 2021). Rural tourism associations, in particular, have provided a space for the exchange of experiences and knowledge for the sector and where promoters have found opportunities to participate in specific training for the activity (Petridou and Glaveli 2008).

The study of networking in rural tourism during two different economic crises, one in 2008 (pre Covid period) and the recent one caused by Covid-19 pandemic, is the focal point of this research, parting from the idea of identifying gender-based differences in promoters’ attitudes and opinions towards it on the island of La Palma. More specifically, this study will be looking at tourism promoters’ involvement in the sector’s associations, the importance they give to networking, access to training and its relation to associating, and the relationship between institutional support and promoters’ associative behavior. Finally, from a gender perspective, we will also study the importance of networking as perceived by the advantages and disadvantages it involves. In fact, there are studies in the literature analysing people’s involvement in tourism activities during the Covid pandemic (Brouder 2020; Roman et al. 2020; Vinerean et al. 2021), but not referring to the specific case of rural tourism and also including the study of variables related to business management and gender. Among the variables related to business management considered in this study are specific training for carrying out tourism management activities and the processing of subsidies for the start-up of tourism activities by promoters. Similarly, in the literature we also find studies that relate networking to rural tourism in the post Covid period (Mirakzadeh et al. 2021; Peira et al. 2021), though they do not analyse the relationship between networking and gender after the pandemic. Thus, for example, in the study carried out here, it is observed that associationism is preferentially related to the male gender and specific training to the female gender.

## Literature review and hypotheses development

### Networking in rural tourism

Using a systemic approach to the analysis of rural tourism, this section looks in depth at the set of relationships between the actors involved in offering tourism activities (Tirado Ballesteros and Hernández Hernández 2019; Gispert and Clavé 2020).

Rural tourism is an activity that is constructed socially through the participation of various actors: individuals and groups that are involved in the activity according to their own set of idiosyncrasies and the specific needs of each place (Verbole 2003; Schroeder et al. 2016). The networks, interactions, and partnerships between public and private actors set the path for rural tourism to follow (Cánoves et al. 2004).

Moreover, it is a must for actors to conform to these networks to face the structural atomization usually found in the countryside. In fact, in rural environments, due to their sociodemographic and territorial circumstances, there is a certain propensity for family businesses to form (Anthopoulou 2010; Bessière 2014).

Additionally, progress in rural tourism has not only been influenced by family ties, but by the participation of promoters in different local groups based on their values and loyalties as well (Verbole 2003). Depending on the number and type of relations that an actor may have in the networks, they will participate in the inclusion or exclusion process that can ease or hinder their access to resources, information, and subsequently, the realization of their interests in the rural tourism market.

The informal networks between promoters within rural tourism have proven to be relevant for a considerable number of businesses who have entered the market once becoming aware of other local entrepreneurs’ success. This results in more people mimicking the decision to start a business in rural tourism (Francès 2007). The knowledge and interactions with promoters produce confidence and security to undertake such an activity, in a way that other nodes next to it, in the network, kickstart other entrepreneurial efforts in the field.

Moreover, to assure small businesses’ sustainability in rural tourism, it is essential for them to collaborate with other actors, since many of the resources and qualifications needed are not available for each new start-up. Formal and institutional networks (such as public administration and rural tourism associations) can also support and boost this activity by alleviating any uncertainties with information, assessment, and specific training for promoters in rural tourism (Petridou and Glaveli 2008).

### The impact of the crisis on rural tourism networking

Fotiadis et al. (2021) forecast international tourist arrivals from July 2020 to June 2021, including three recent crises and concluded that the drop in tourist flows ranges from 30.8 to 76.3%. However, Beridze et al. (2020) predicted that rural tourism would be more popular after the COVID-19 pandemic, as tourists would tend to avoid crowded places. For example, the study carried out by Jeon and Yang (2021) examined the structural changes of a local tourism network focusing on the Gangwon Province, in the Republic of Korea, which experienced a rise in tourist demand following the COVID-19 outbreak. The study focused on the movement patterns of tourists who visited the province during periods before and after the outbreak, finding as the main result that tourists focused their movements on local areas.

At the time of the COVID pandemic, one of the factors that influenced the organization of tourism activities was the social one, and particularly the co-participation-related strategies (Roman et al. 2020; Brouder 2020). In this respect, Peira et al. (2021) afirmed that due to the COVID-19 pandemic, in the tourism market, perceptions regarding the importance of local networks have developed as well as their specific characteristics that facilitate and consolidate the creation of local stakeholder’ networks. In the particular case of Mirakzadeh et al. (2021), their research focused on the analysis of preventive behaviour of rural tourism hosts in the face of COVID-19 pandemic. Specifically, recommendations by regional health experts and other local social networks in the region and villages as cues to action were highlighted.

Gabriel-Campos et al. (2021) studied community eco-tourism in rural Peru. They concluded that further development and implementation of appropriate risk management strategies to counteract climate change and enhance the community's resilience of its eco-tourism system were need to sustain organizations’ wider social networks, of which the community is a part. Importantly, they affirm that this finding might be relevant to other local communities seeking to improve their resilience to COVID-19 and climate change.

Finally, as an example of a specific management strategy to be appied, Purwaningsih et al. (2021) studied ecollaboration for rural tourism recovery after COVID-19. They explored the opportunities, strategies and barriers to ecollaboration in tourism business activities, and found that the effects of the pandemic encouraged ecollaboration and accelerated the recovery of tourism. Likewise, during the study of Peira et al. (2021), the creation of stakeholder’ networks, and digital transformation were the most critical issues which emerged. For instance, farmers stressed the need to strengthen both collaboration among themselves and relations with other stakeholders.

However, despite the diversity of studies on rural tourism, we have not found any in the literature that analyse the situation before and after the COVID crisis, from the perspective of the importance given to associationism, while also considering the role of women in the promotion and development of tourism activity.

### Hypotheses development

In terms of networking in rural tourism, the leading role of women has favored their participation in its formal and informal networks. As a result, women have access to the benefits of mutual support in addition to the social and economic recognition of their contributions. Despite this, although women occupy technical positions in associations’ administration, their governing and executive bodies are represented almost entirely by men. This showcases both the lack of female public protagonism and gender inequality in the design and administration of the initiatives promoted by rural tourism associations (Szmulewicz et al. 2012).

Regarding the importance given to association, women have a tendency to value it more positively than men. A possible explanation for this better consideration that women have of associations could be that they offer a support network for their traditional social and family duties, so that they can better reconcile their personal and professional lives (Gentry 2007; Prince 2017). Actually, family businesses in rural tourism have continued to display the traditional gender roles in terms of labor distribution. The human capital available for families has been organized in a way that perpetuates the domestic role of women in these new rural niches. In doing so, the jobs of rural women in family businesses are linked to the household occupations they have traditionally taken care of: cleaning rural houses, cooking, and caring for visitors among other tasks (Cánoves et al. 2004; Anthopoulou 2010; Brandth and Haugen 2010; Dhanaraj and Mahambare 2019)

Nevertheless, their participation in associations is typically unrelated to feminist principles that demand social change. Quite the opposite: some studies show that the main reason for women to engage in associations is to socialize more than they would in the domestic space (Movono and Dahles 2017). In this sense, even though there is no explicitly feminist motivation, female networking favors the public presence of women, which in turn gives them access to the networks and information necessary for their independence from traditional gender roles. Therefore, associations can act as “bridges” (Pallares-Barbera and Casellas 2019) for women to reach equality by collectively becoming aware of oppressive situations (Oldrup 1999), and thus opt for changes that can improve their quality of life.

From another perspective, women’s participation in the third sector could also be tied to the acts of service that they have traditionally performed. Themudo (2009) argues that the willingness women have to cover the needs that governmental institutions do not is linked to female empowerment in the sense that it promotes their participation in the community’s social capital (Anggadwita et al. 2021). This is why, despite mostly carrying out traditionally female activities, their participation in third-sector organizations gives them access to more job sources (Taniguchi 2006) and information while also receiving greater recognition for their social contributions. Active female participation favors their empowerment because in doing so, the protagonistic roles they adopt can lead to the necessary personal and public progress towards their emancipation (Movono and Dahles 2017). Therefore, the quality of their connections through these networks directly correlates to the opportunities they will have to improve their personal and social life (Prince 2017).

Nonetheless, the responsibilities derived from women’s family and occupation can hinder work related to volunteering and networking, because they involve a greater commitment in terms of their time management relative to men’s (Taniguchi 2006; Osborne et al. 2008; Özbilgin et al. 2011).

In this paper, all the previous approaches are assumed in studying the networking in rural tourism areas. Based on the previous bibliographical study, the following objectives were set to guide the empirical work of this study:

1. Identify the importance of networks in rural tourism activity and highlight differentiated behaviours of networking according to gender.
2. Recognise the role of the public administration in promoting the generation of networks in rural tourism.
3. Determine the relationship between access to social capital and provision of knowledge and training in rural tourism.
4. Evaluate perceptions regarding the advantages and disadvantages of networking in rural tourism by the promoters of the activity.
5. Understanding the behavior of rural tourism promoters towards networking during the 2008 and Covid-19 crises.

In terms of the empirical subject of this research, the main hypothesis we postulate is that `there are differentiated behaviours between male and female rural tourism promoters towards networking during the 2008 and Covid-19 crises´. In particular, the following are the specific research questions posited by this research: is there a favourable predisposition towards networking on the part of rural tourism promoters; do women tourism promoters value more positively networking in rural tourism than men promoters; are women more involved than men in rural tourism organisations; has the institutional support given to rural tourism enterprises favoured the association of promoters in this activity; does the association of rural tourism promoters encourages the transfer of knowledge about the activity; and finally, how do tourism promoters perceive the impact of the Covid-19 on rural tourism?

Specifically, the hypotheses of the study has been formulated as follows:

#### H1

 After Covid, promoter profile changes in terms of gender.

#### H2

H2: Associationism increases after Covid in both men and women.

#### H3

After Covid, there is an increase in specific training of promoters, both men and women.

#### H4

Being associated guarantees the promoter greater access to specific training, both for men and women.

#### H5

Being associated, the promoter receives more subsidies than if he/she were not a partner, both for men and women.

#### H6

Getting specific training increases the chances of getting a subsidy from the promoter, both for men and women.

#### H7

After Covid, the pros and cons of associationism in rural tourism are valued in a similar way as before Covid.

#### H8

The pros and cons of associationism in rural tourism are rated similarly by both men and women.

#### H9

The pros and cons of associationism in rural tourism are valued in a similar way, both by associated and non-associated promoters.

#### H10

The impact of Covid on rural tourism is perceived in a similar way by both men and women.

#### H11

The impact of Covid on rural tourism is perceived similarly by both associated and non-associated promoters.

## Method

To test the above hypotheses, we have carried out a quantitative study for which a survey has been the main tool to gather information. The fieldwork for this research took place in the small island of la Palma, on the rural tourism accommodation establishments and their promoters, twice in 2007 and 2021. Afterward, a descriptive analysis of the collected data was developed, making use of the hypothesis tests analysis in order to study the advantages and inconveniences of networking in rural tourism as perceived by promoters. In addition, Logistic Regression Models have been used.

### Information collection and the population

This research focuses on the study of the rural tourism offer on the island of La Palma. Based on the official register of rural tourism establishments from the Department of Tourism and Transport of the Island Council of La Palma, a total of 195 rural houses were identified in the island in 2007, with 234 accommodation units and 829 tourist places.

Therefore, rural tourism accommodation establishments and the promoters that manage them constitute the empirical subject of this study. This led to a total of 95.6% of promoters and 92.8% of rural accommodations being consulted from the Island Council of La Palma’s official register. The study consisted, in a first stage, of a questionnaire answered by 154 promoters for 181 rural accommodations with a total of 219 housing units between February and May 2007 (Pre Covid-19). This sample is highly representative, since it covers of the promoters, making it almost a complete census.

On the other hand, to measure the impact that the Covid-19 pandemic has had on rural tourism in La Palma, a random sample of 81 of previously interviewed promoters was selected (the necessary sample size being at least 63, for a maximum estimation error 10% and under the dichotomous hypothesis p = q = 0.5). These promoters were interviewed by telephone from March to September 2021 (post Covid-19). This enabled us to verify that only 43.2% of them continued to devote their housing to rural tourism.

The questions included in the questionnaire were discussed by the authors and with industry practitioners**.** Data were collected through face-to face meetings with rural tourism promoters, either in their homes or their rural houses or other agreed establishments where the questionnaire was filled in. Prior to the personal interview, the time and place were confirmed by a telephone call, as well as the presence of the main tourism promoter. Finally, it should be added that questions were aimed mainly at finding out the opinions and personal details of promoters of rural tourism accommodation. Thus, it was essential to meet the main people in charge of managing the rural tourism activity.

The questions were closely linked to the clear delimitation of the objectives of the research, as well as the topics of interest (Cuervo Arango 1993: p. 264). The questionnaire incorporated open and closed questions, including those with scale answers.

The post Covid questionnaire was carried out by telephone in 2021 and included similar questions to those asked in the face-to-face questionnaires of 2007, incorporating various questions related to the impact of the social and health crisis derived from Covid-19 on rural tourism activity on the island of La Palma. The questionnaire was given in Spanish and then translated into English by a professional translator to incorporate its questions into this article.

First, the list of people who had been contacted in the 2007 questionnaire was used for the telephone questionnaires. Interviewers began by identifying the respondent as either the owner, the manager of a rural tourism establishment or another person involved. They were then asked if the establishment was currently being offered to visitors. If the answer was negative, the respondent was asked to mention the main reasons why he/she had not continued to offer their rural house for tourism purposes. This was a multiple-choice question, which allowed a choice of several reasons for not continuing to offer rural tourism. The reasons to choose were the following:

- Working or busy with other tasks, with no time for the house.
- It is not profitable, due to the maintenance required.
- Because of other family needs e.g. to occupy the house for children, relatives, etc.
- There is no generational replacement (child or family member to take over).
- Because of the Covid situation: occupancy has dropped considerably.
- Because of the Covid situation: fear of contagion.
- Personal issues—old age, illness, death, family care, etc.
- Because property has been offered for long-term rent—fewer problems and fixed rent.
- Due to sale of property.
- Other

For the group of people who were not currently continuing to offer rural tourism accommodation, this was the end of the telephone questionnaire.

For those who responded that they were continuing with the activity, they were asked about the main reasons for offering rural tourism. This was also in a multiple-choice format incorporating the following reasons:

- It is a source of income -necessary or complementary.
- It is a professional occupation in rural tourism management.
- There is no other occupation, being unemployed, or other compatible activities being carried out.
- It is a source of personal fulfilment.
- To preserve the heritage and prevent its deterioration.
- To avoid problems such as squatting, theft, etc.

Next, they were asked about the degree to which they were affected by the Covid situation with respect to their rural tourism activity, asking them to evaluate on a scale of 1–5, where 1 meant that they had not been impacted at all, and 5 meant that they had been greatly affected by the situation.

Subsequently, they were asked if they had received specific training related to rural tourism, or if they had participated in meetings for analysis and debate, such as courses, seminars, talks or training trips, with a dichotomous Yes/No answer to this question. In addition, they were asked if at any time they had received any subsidy for the implementation and development of their rural tourism activity, with a dichotomous answer of Yes/No. In turn, the respondents were asked if they were part of an association to promote or manage their rural tourism house, with a dichotomous answer of Yes/No. These three questions were asked in the same way in the questionnaire prior to the Covid situation in 2007.

Next, several statements had to be rated on a scale of 1–5, with 1 not agreeing at all and 5 strongly agreeing with the statement:

- Rural tourists will be more sensitive to health aspects after the Covid crisis.
- Rural tourists will be more sensitive to environmental and climate change issues after the Covid crisis.
- Managing individually, without an association, rural accommodation is more difficult because of the Covid crisis.
- Rural tourism has been less affected than the rest of tourism by the Covid crisis.
- The Covid situation has had a favourable influence on my interest in associationism.
- Rural tourism could be an option for the future, after Covid, due to the interest of tourists in seeking isolation, nature and less overcrowding.
- Booking platforms-booking, Airbnb-will have more weight in generating occupation in rural tourism after Covid.
- The Covid crisis has forced prices in rural accommodation to fall.
- Direct public subsidies to rural accommodation is essential to cope with the loss of income caused by Covid.
- Specific promotion of the values of rural tourism is needed to differentiate Covid in the current crisis.

Subsequently, respondents were asked to give their opinion on different aspects related to rural tourism associations, again on a scale of 1–5, with 1 being totally disagree and 5 totally agree. Firstly, they were asked to consider the degree of importance that associations have had on the competitiveness of rural tourism on the island. Then, they were asked to rate their assessment of associationism in rural tourism, according to the following advantages and disadvantages, which coincided with the same advantages and disadvantages asked in the questionnaire prior to Covid:

- It helps improve the quality of the offer.
- It unifies the image of the product.
- It facilitates joint promotion.
- It facilitates access to subsidies.
- It simplifies the management of reservations.
- It is a bureaucratised and unwieldy system.
- It restricts individual freedom -prices, promotion, etc.
- Being a member increases the possibilities of receiving help to deal with Covid -training, subsidies….

A final section offered the possibility of expressing recommendations or suggestions in relation to the situation of the rural tourism offer and associationism in the activity in the times of Covid, in an open-ended question.

Finally, data were collected to obtain the sociodemographic profile of the respondents, such as gender, age and level of studies, aspects also measured previously in the pre Covid questionnaire.

### Sociodemographic features of rural tourism promoters in La Palma

Before analysing the results in detail, it is very important to stress that after Covid, 47.4% of the promoters continued with their rural tourism activity, hence only 81 of the 154 pre Covid promoters were interviewed.

In La Palma in the period prior to Covid-19 (year 2007), from a total of 154 promoters, there were 87 men and 67 women, which in terms of percentages are 56.5% male and 43.5% female, respectively. The larger proportion of men who decide to take part on this entrepreneurial endeavor on the island is consistent with the traditionally low presence of women in business, especially in rural environments. Nevertheless the situation changes considerably for the post Covid19 period. In the post Covid-19 sample of promoters, there were 37 men (45.7%) and 44 women (54.3%).

Nonetheless, it is also true that entrepreneurship in rural tourism accommodation does not necessarily mean that the promoter is the only person in charge of the activity. Considering that these are generally family businesses, the domestic duties necessary for the activity, along with the functionalities tied to each sex that generally prevail in rural environments, it is not out of place to assume that women lead a more active role in the daily management of rural tourism.

Regarding the ages of La Palma’s rural tourism promoters in 2007, the majority (76%) fall into the categories of 36–50 and 51–65. To this percentage, there is another 17% that could be added, consisting mainly of promoters older than 65. In the post covid19 period, regarding their current age, we can say that while 47.5% are between 51 and 65 years old, a high percentage (40.7%) reach or exceed 65 years.The profile in question is therefore of adults dedicated to rural tourism, which fits with the trend of populational aging in rural environments during the last decades.

In the following table we can appreciate the sociodemographic and activity’s variables description.

### Analysis techniques

The empirical work uses quantitative research techniques as methods of collecting primary information. Using this quantitative approach, data collection was based on the measurement of the variables or concepts derived from the hypotheses (Hernández Sampieri et al. 2006). Therefore, the phenomenon studied, in this case, networking in rural tourism on the island of La Palma, from a gender perspective, must be measurable (Gispert and Clavé 2020). In addition, the analysis of the data collected by the questionnaires was carried out using the statistical tool SPSS (IBM Corp. 2019).

Data analysis has been carried out from a gender perspective, distinguishing general trends from specific trends according to gender. To do this, the data have been segmented between men and women rural tourism promoters for each of the variables studied. Furthermore, reference should be made to the statistical significance of the data collected. In this study, to find data with adequate statistical significance, an error level of 0.05 was used (Denis 2018).

Regarding the analysis techniques, firstly, descriptive statistics have been used to analyse the variables related to the Covid effect. As an indicator of the relationship between the variables, since at least one of them is nominal, the Chi-square coefficient has been determined, as well as the corresponding significance or p-value. These measurements appear in Table 1 and allow us to verify hypothesis H1, (and H2 and H3 in general, these two not by gender).

**Table 1:** Sociodemographic and activitiy’s variables

	Pre covid freq. (%)	Post covid freq. (%)	Chi-square	*p*-value
*Gender*			2.491	0.095*
Male	87 (56.5)	37 (45.7)		
Female	67 (43.5)	44 (54.3)		
*Age*			31.337	0.000**
26–35	10 (6.5)	0 (0.0)		
36–50	58 (37.7)	9 (11.3)		
51–65	60 (39.0)	38 (47.5)		
Over 65	26 (16.9)	33 (41.3)		
*Educational level*			10.274	0.016**
None or incomplete primary education	19 (12.3)	17 (21.3)		
Primary education	54 (35.1)	13 (16.3)		
Secondary education	43 (27.9)	25 (31.2)		
University education	38 (24.7)	25 (31.2)		
*Degree of importance that associationism has had on the competitiveness of rural tourism*			4.277	0.370
Very low	3 (2.0)	2 (5.7)		
Low	10 (6.6)	0 (0.0)		
Medium	29 (19.1)	7 (20.0)		
High	47 (30.9)	13 (37.1)		
Very high	63 (41.4)	13 (37.1)		
*Member of association to promote or manage your rural tourism establishment*			3.826	0.050*
Not associated	81 (52.6)	28 (34.3)		
Associated	73 (47.4)	53 (65.7)		
*Specific training on rural tourism*			11.266	0.001**
Not received	79 (51.3)	16 (20.0)		
Received	75 (48.7)	65 (80.0)		
*Subsidy to develop rural tourism activity*			3.831	0.147
Not received	44 (29.4)	14 (17.4)		
Received	106 (70.6)	67 (82.6)		

Samples sizes: pre covid (*n*_1_ = 154) and post covid (*n*_2_ = 81)

*p < 0.10: p-value < 0.10

**p < 0.05: p-value < 0.05

**Fig. 1:**
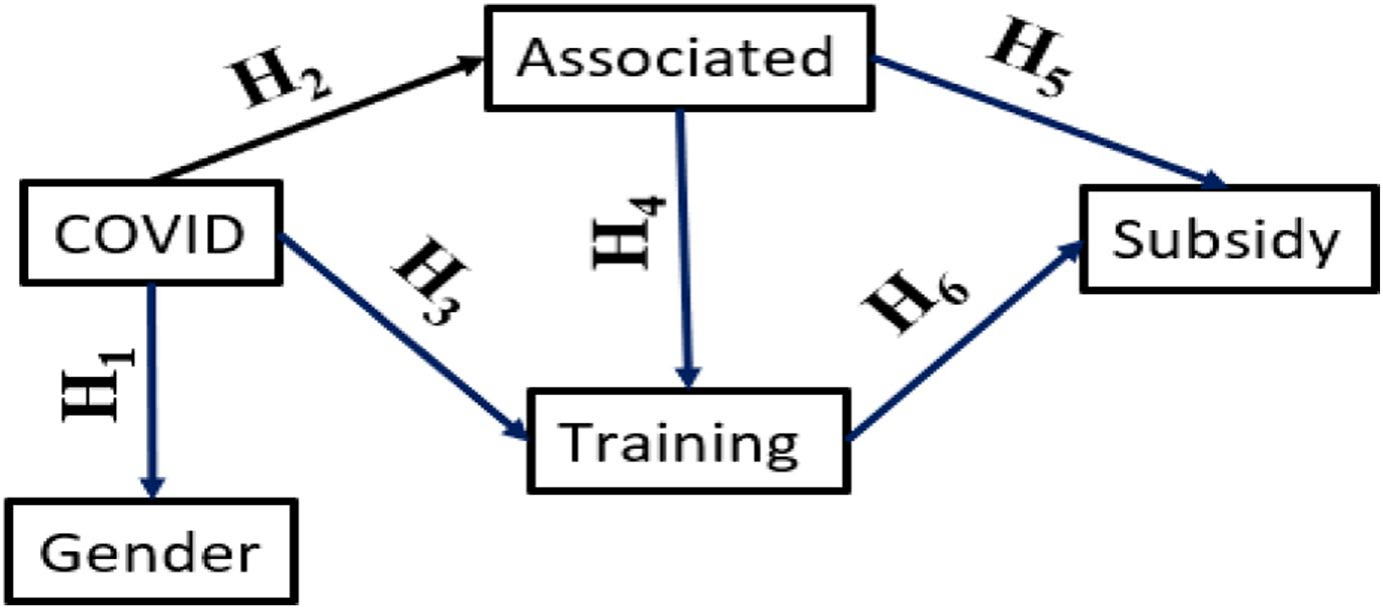
Proposed research model with causal relationships between the variables

**Fig. 2:**
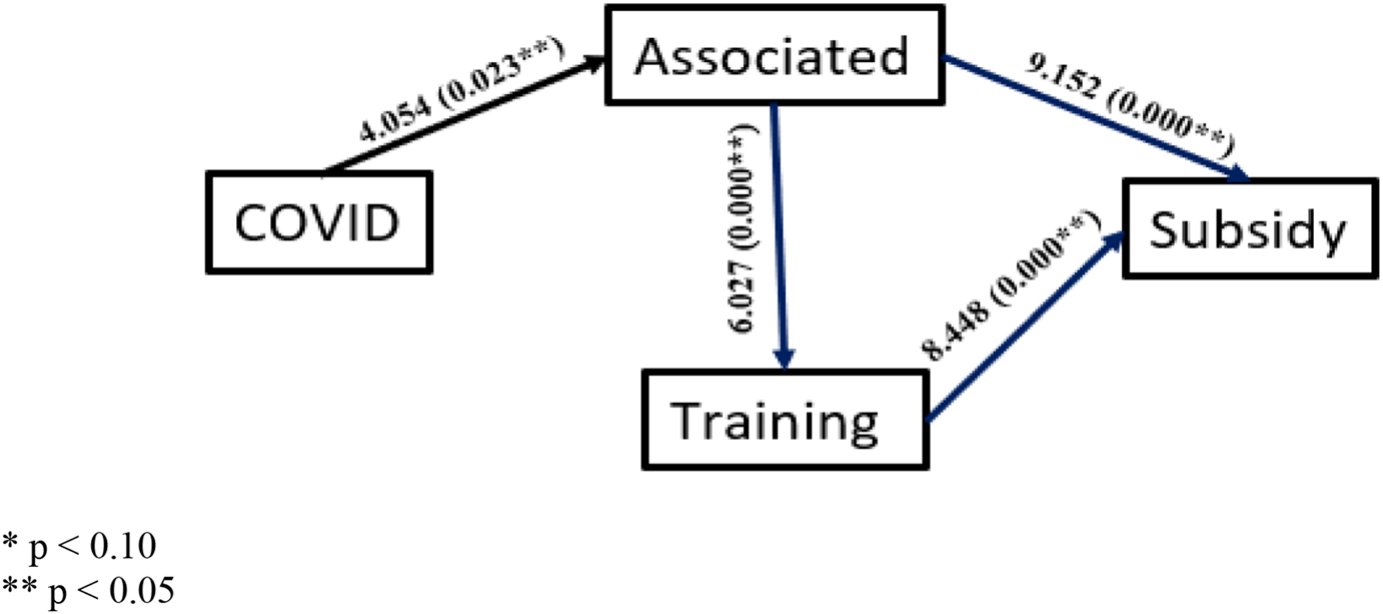
Logistic regressions (odd ratios and *p*-values) for male promoters

**Fig. 3:**
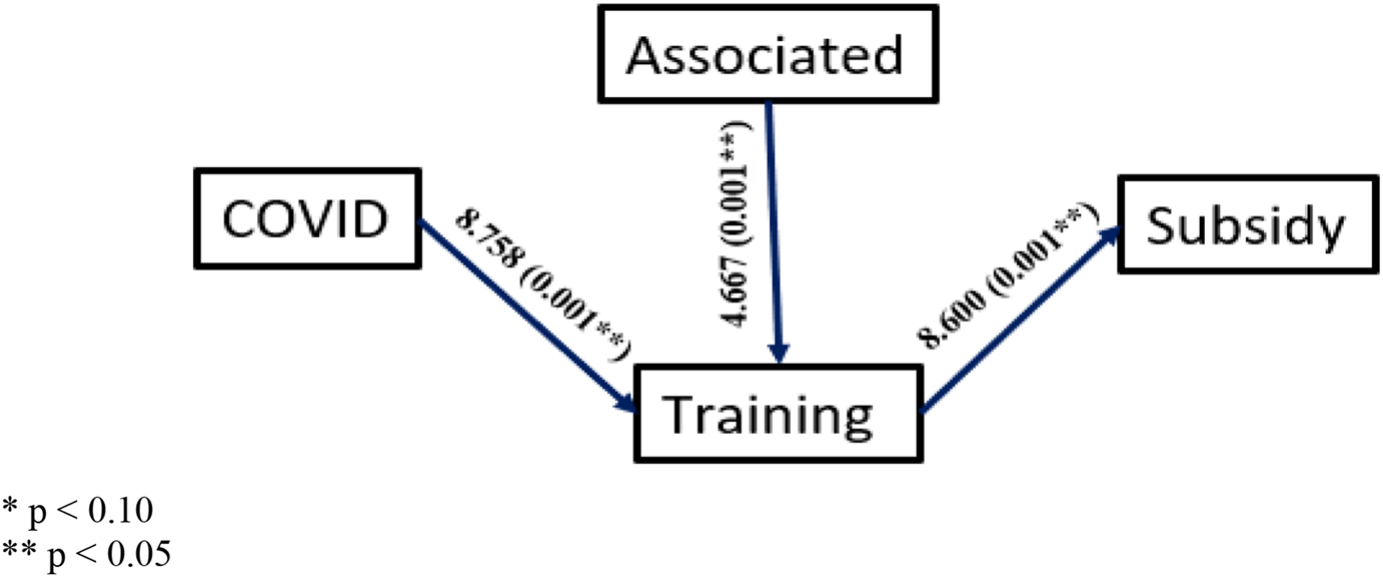
Logistic regressions (odd ratios and *p*-values) for female promoters

In addition, to verify hypotheses H2-H6, Logistic Regression Models have been used to check if the influence of each independent variable on the dependent variable is significant at every stage of the model proposed in Fig. 1. This analysis has been carried out from a gender perspective, distinguishing specific trends according to gender. To do this, the data have been segmented into men and women rural tourism promoters, obtaining the results shown in the Figs. 2 and 3 (where the Odd ratio and signification or p-value for each pair of variables are analysed).

Finally, we have tested hypotheses H7–H11 by comparing the Covid effect with Gender and being a member of an association. The average rating of promoters’ of the advantages, disadvantages and other statements related to the effect of the pandemic on being in a rural tourism association was tested using a nonparametric test like Mann–Whitney. The results appear in Table 3.

## Results

As a starting point and analysing Table 1, we conclude that Covid (pre and post) has a significant influence on Gender (0.095*), Age (0.000*), Educational Level (0.016**), Associated (0.050*) and Training (0.001*). The results with respect to age are normal given that they are the same promoters but older.

In relation to sociodemographic and activity variables, Table 1 shows that after Covid, the profiles of promoters change in terms of gender, thus H1 is fulfilled. For example, the presence of women promoters increases. Second, associationism increases after Covid, although this is true only for men (Figs. 2 and 3). The level of specific training of promoters also increases, though this is confirmed only for women (Figs. 2 and 3).

The effects of being an associate vary as follows. First, being associated guarantees the promoter greater access to specific training, both for men and women (Figs. 2 and 3). In addition, being associated, the male promoter receives more subsidies than if he were not associated (Figs. 2 and 3).

Figures 2 and 3 show the causal relationships that are significant, indicating in each case both the value of the Odd ratio and the significance or *p*-value corresponding to the logistic regression model obtained for each pair of variables. The Odd ratio indicates the change in the quotient between the probability that the dependent variable (dichotomous) takes the value 1 and the probability that it takes the value 0 when the value of the independent variable is increased by one unit, so that an OR = 1 would indicate that there is no variation and therefore no relationship between the two.

After comparing Figs. 2 and 3, we find that there are causal relationships that are confirmed as significant in both genders, as is the case for H4 and H6, and others that are partially confirmed as significant, i.e. in one of the two genders, as is the case for H2, H3 and H5. These provide relevant conclusions when it comes to justifying the influence of gender on the set of variables that we consider when studying associationism: being associated, subsidies and training.

H2 and H5 are confirmed only in the case of men. First, the Covid effect has significantly influenced being associated. In this case, as OR = 4.054, we can conclude that after Covid, the probability of a (male) promoter being associated (versus not being associated) is 3.054 times higher than before Covid. Second, being an associate has a significant influence on obtaining a subsidy. Here, since OR = 9.152, we conclude that the probability of a (male) promoter obtaining a subsidy (as opposed to not obtaining a subsidy) is 8.152 times higher being an associate.

In relation to H3, we find that it is only confirmed in the case of women. The Covid effect had a significant influence on receiving specific training. In this case, we highlight the OR value = 8.758, which allows us to conclude that, after Covid, the probability of a (female) promoter having received such training (as opposed to not having received it) is 8.758 times higher than before Covid.

On the other hand, H4 and H6 are confirmed for both genders. First, being an associate has significantly influenced receiving a specific subsidy, with the probability of receiving a subsidy (vs. not receiving a subsidy) being 5.027 and 3.667 times higher for men and women, respectively, when being associates.

Second, having received specific training has a significant influence on obtaining a subsidy, with the probability of obtaining a subsidy (compared to not obtaining a subsidy) being 7.448 and 7.600 times higher for promoters who have received specific training.

### Assessment of the pros and cons of associationism

To measure the quality of the pros and cons questionnaire, we focus on two aspects: reliability and validity. Firstly, to assess the reliability of the questionnaire, we carry out an analysis of its internal consistency, which is measured through Cronbach's α coefficient, whose value must exceed 0.7 to ensure that the items all measure the same concept or construct (Cronbach 1951). This process also offers the possibility of excluding those items that contribute the least.

In our case, we focused on the reliability of the questionnaire formed by the items included in Table 2, both in the pre covid stage (first 7 items, advantages and disadvantages) and in the post covid stage, in which, in addition to the above, the questionnaire includes the remaining 10 items (statements). In both cases, realiability is confirmed for the total of the 17 items, since Cronbach's α coefficient values of 0.788 and 0.713 were reached, respectively.

**Table 2:** Adventages/disadvantages of associationism and statements of the impact of the covid on rural tourism

ADV1: Unifies product image	
ADV2: Encourages joint promotion	
ADV3: Helps to improve the quality of the offer	
ADV4: Facilitates access to foreign markets	
ADV5: Simplifies the management of bookings	
DIS1: It is a bureaucratic and unwieldy system	
DIS2: Restricts individual freedom (prices, promotion, etc.)	
STA1: Rural tourists will be more sensitive to health aspects after the Covid crisis	
STA2: Rural tourists will be more sensitive to environmental and climate change issues after the Covid crisis	
STA3: Individual (non-associated) management of rural accommodation is more difficult because of the Covid crisis	
STA4: Rural tourism has been less affected than the rest of tourism by the Covid crisis	
STA5: The Covid situation has had a favourable influence on my interest in associationism	
STA6: Rural tourism could be an option for the future after Covid due to the interest of tourists in seeking isolation, nature and less overcrowding	
STA7: Booking platforms (Booking, Airbnb, etc.) will have more weight in generating bookings in rural tourism after Covid	
STA8: The Covid crisis has forced me to lower prices of my rural accommodation	
STA9: Direct public subsidies to rural accommodation are essential to cope with the loss of income caused by Covid	
STA10: A specific promotion of the values of rural tourism is necessary to differentiate it following the Covid crisis	

Regarding the changes in the assessment of the pros and cons of associationism, as shown in Table 3, in general, the pros are considered to be quite important for promoters with most of the ratings being 4 out of 5 (High). However, as for the cons, the promoters do not consider them as important, with ratings ranging from 2 (Low) to 3 (Medium). Perhaps the most important pros are "Unifies product image", "Encourages joint promotion" and "Simplifies the management of bookings".

**Table 3:** Exploratory factorial analysis in rural tourism, pre and post covid

Post covid (17 items)			Pre covid (7 items)	
Cronbach’s *α* = 0.713			Cronbach’s *α* = 0.788	
KMO = 0.721	Bartlett’s *p*_value = 0.000		KMO = 0.837	Bartlett’s *p*_value = 0.000
*Items*	Factor loading	Cumulative variance	Factor loading	Cumulative variance
*Factor 1: Advantages*		24.844%		48.141%
*ADV1*	0.860		0.839	
*ADV2*	0.756		0.807	
*ADV3*	0.794		0.831	
*ADV4*	0.766		0.846	
*ADV5*	0.570		0.712	
	AVE = 0.571	CR = 0.867	AVE = 0.654	CR = 0.904
*Factor 2: Disadvantages*		40.608%		72.687%
*DIS1*	0.919		0.856	
*DIS2*	0.827		0.800	
	AVE = 0.764	CR = 0.866	AVE = 0.686	CR = 0.814
*Factor 3*		52.898%		
*STA1*	0.773			
*STA2*	0.797			
*STA10*	0.644			
	AVE = 0.549	CR = 0.784		
*Factor 4*		61.540%		
*STA3*	0.713			
*STA5*	0.598			
*STA6*	0.829			
	AVE = 0.518	CR = 0.760		
*Factor 5*		69.273%		
*STA4*	0.781			
*STA7*	0.695			
*STA8*	0.675			
*STA9*	0.678			
	AVE = 0.502	CR = 0.801		

Rotation: VARIMAX

Regarding H7 and H8, the information in Table 3 shows that, on the one hand, the Covid effect significantly influences the advantage "Facilitates access to foreign markets" and the disadvantage "It's a bureaucratic and unwieldy system". By gender, we find differences in the advantage "Simplifies the management of bookings" for women and in two disadvantages, "It's a bureaucratic and unwieldy system" and "Restricts individual freedom (prices, promotion, etc.)" for men.

However, with respect to H9, Table 3 indicates that there are significant differences in the average ratings of all advantages and disadvantages by associates and non-associates. Associated promoters assign more importance to each of the advantages than non-associates, while they assign less importance to the disadvantages than non-associates. Therefore, H9 is rejected. This result, although logical, is quite relevant as it demonstrates a significant change in impression or perspective depending on whether the promoter is associated or not.

Secondly, we analyse the validity of the questionnaire (Appendix 1) according to the following criteria: construct and convergent validity. For this purpose, we conduct an Exploratory Factor Analysis (EFA) using the factor component method and Varimax rotation to facilitate the interpretation of the coefficients. The results obtained are shown in Table 3.

The purpose of construct validity is to check whether the relationships between the items of the questionnaire guarantee the construction of an adequate dimensional structure, which can then be extended to other populations. Looking at the results in Table 3, we notice that the Kaiser–Meyer–Olkin sample adequacy coefficient, KMO, presents values above 0.7, 0.837 and 0.753, respectively, which guarantees that there is an internal correlation between the items that is significant enough to extract the factors or constructs, both for the pre Covid and the post Covid tourism promoters. This condition is confirmed by the significance of 0.000 obtained in Bartlett's sphericity test. Therefore, it makes sense to further interpret the analysis, noting that, in both cases, two factors or constructs are generated to explain the advantages (factor 1) and disadvantages (factor 2). Moreover, in the post Covid case, 3 additional factors are generated to collect the 10 statements, which, when combined with the first two, manage to explain 69.27% of the total variance, very close to the 72.69% obtained in the pre Covid case.

In addition, we analyse the convergent validity of the questionnaire through two indicators: the average variance extracted, AVE, which is the sum of squared factor loadings divided by the number of items, and the composite reliability, CR, which is calculated as the sum of squares of factor loadings divided by this sum plus total of variance of the error term for each item. The values of both coefficients are shown in Table 3. As both factors present AVE values higher than 0.5 in both pre Covid and post Covid contexts, we can conclude that all constructs present a high explained variance, thus confirming their validity (Chin et al. 1998). Moreover, since the obtained values of CR are greater than 0.7, we also guarantee that, in both cases, the extent to which the items are related to each other within each construct or factor is quite adequate (Hair et al 2017) (Table 4).

**Table 4:** Adventages/disadvantages of associationism and statements of the impact of the covid in rural tourism pre and post covid, by gender and member of association

	Pre covid (Mean)	Post covid (Mean)	Mann–Whitney (*p*-value)	Male (mean)	Female (mean)	Mann–Whitney (*p*-value)	Not associated (mean)	Associated (mean)	Mann–Whitney (*p*-value)
*ADV1*	4.03	4.17	0.683	4.00	4.12	0.775	3.57	4.51	**0.000**
*ADV2*	4.18	4.00	0.186	4.17	4.13	0.407	3.70	4.57	**0.000**
*ADV3*	3.87	3.94	0.683	3.80	3.98	0.342	3.34	4.40	**0.000**
*ADV4*	4.11	3.57	**0.006**	3.99	4.04	0.842	3.76	4.25	**0.003**
*ADV5*	4.05	4.12	0.709	3.91	4.23	**0.040**	3.41	4.68	**0.000**
*DIS1*	2.91	2.37	**0.031**	2.98	2.60	**0.043**	3.33	2.31	**0.000**
*DIS2*	2.64	2.46	0.514	2.80	2.38	**0.034**	3.25	2.00	**0.000**
*STA1*	–	4.03	–	4.13	3.95	0.410	4.00	4.05	0.958
*STA2*	–	3.94	–	3.94	3.95	0.806	4.00	3.91	0.694
*STA3*	–	3.83	–	3.88	3.79	0.935	3.33	4.09	**0.062**
*STA4*	–	3.63	–	3.56	3.68	0.545	3.83	3.52	0.644
*STA5*	–	2.09	–	2.44	1.79	**0.076**	1.58	2.35	0.123
*STA6*	–	4.34	–	4.44	4.26	0.731	4.25	4.39	0.482
*STA7*	–	3.54	–	3.69	3.42	0.441	3.50	3.57	0.797
*STA8*	–	2.89	–	2.88	2.89	0.961	2.83	2.91	0.905
*STA9*	–	4.09	–	4.13	4.05	0.987	4.33	3.96	0.503
*STA10*	–	4.40	–	4.38	4.42	0.987	4.42	4.39	0.959

*p < 0.10: p-value < 0.10

**p < 0.05: p-value < 0.05

### Perception of Covid's impact

As for how the promoter perceives the impact that Covid has had on rural tourism, we highlight that, based on the data (from the last 10 rows) of the post Covid column of Table 3 and using the Wilcoxon test for two-by-two comparisons, the following four homogeneous groups of indicators would be formed. These are listed in increasing order, according to their impact for the promoter: G1 = {STA5}, G2 = {STA4,STA8}(*p* = 0. 183), G3 = {STA1,STA2,STA3,STA9} (*p* = 0.572) and G4 = {STA6,STA10} (*p* = 0.828). Therefore, we can interpret that ‘Rural tourism could be an option for the future after Covid because of the interest of tourists in seeking isolation, nature and less overcrowding’ and ‘A specific promotion of the values of rural tourism is necessary to differentiate it following the Covid crisis’ are the indicators that matter most to promoters. While ‘The Covid situation has had a favourable influence on my interest in associationism’ is the least important followed by ‘The Covid crisis has forced me to lower prices of my rural accommodation’ and ‘Rural tourism has been less affected than the rest of tourism by the Covid crisis’.

Regarding H10 and H11, we can conclude that they are fulfilled except in the case of STA5 by gender and STA3 depending on whether the promoter is associated or not. In the rest of the cases, the impact is considered similar.

### Results and discussion

Specifically, this paper, by analysing the changes due to Covid, has allowed us to determine the relationship between variables of a socioeconomic nature, such as gender, with others more directly linked to business management. In relation to this issue, the results confirm a greater presence after Covid of women promoters in rural tourism activities compared to men. Moreover, it has also shown the greater tendency of women to receive specific training. Likewise, the positive relationship between receiving specific training and being an associate and between obtaining a subsidy for the start-up of the activity and being an associate is maintained with respect to the pre Covid stage, although the latter is only true in the case of men.

The effects of Covid can be seen in the ratings given by the interviewees to the pros and cons of associationism. Thus, we see a more positive evaluation of the advantage: ‘It facilitates access to foreign markets’; while on the other hand, 'It is a bureaucratic and unwieldy system’ is clearly seen as a disadvantage. Based on the previous statements, generally speaking, promoters value networking positively, which demonstrates the benefits they perceive in social capital through covering necessities and achieving lower costs, which is particularly important in rural areas where there is less access to services and resources than urban environments.

By gender, we find differences in one advantage, ‘Simplifies the management of bookings’ for women and in two disadvantages for men; ‘It's a bureaucratic and unwieldy system' and 'Restricts individual freedom—prices, promotion, etc. In reference to women, the support network that associations offer is a way to alleviate the responsibilities traditionally associated with women and helps them reconcile their private and professional lives, as stated in the literature review by Gentry (2007) and Prince (2017). Along these lines, it seems logical that the simplification of booking management appears as the most relevant advantage of networking for women, considering the time they can save with it. Furthermore, associations can become spaces for women to socialize, which is an added benefit to be considered mostly by women, since they have traditionally been assigned to the domestic sphere, especially in rural areas, as Movono and Dahles (2017) held.

Finally, it is important to consider the multidimensional configuration of the advantages of rural tourism associations, considering that every positive aspect studied in this research is statistically significant. In terms of the inconveniences, networking is considered a slow, bureaucratically structured system, which is seen as the result of the institutionalization of formal networks, as opposed to the simplicity and pragmaticism of informal networks or individualized attention. Accordingly, another negative aspect is the considerable restrictions to promoters’ability to act freely. This is probably a result of the traditionally family-run structure of these businesses and the fact that in rural areas they are traditionally managed by individuals, confirming the vision of Verbole (2003) and Schroeder et al. (2016).

On the other hand, no studies have been found in the literature that assess, at the same time, the perception of the impacts of Covid according to the gender of the tourism promoter and whether he/she is a member or not of an association. In this respect, the results obtained in this paper confirm that the perception of the impacts of the Covid crisis does not vary according to gender or whether or not they are members of a rural tourism association.

In summary, no studies by gender have been identified in the literature on associationism in rural tourism that incorporate the changes brought about by Covid, in terms of tendency towards associationism, assessment of its positive and negative effects, access to more specific training for the activity and obtaining subsidies from policy-makers that are necessary for rural tourism activity.

## Conclusions and recommendations

This research has confirmed the initial hypothesis that there are some differentiated associative behaviours in rural tourism based on promoters’ gender when pre or post Covid periods are considered.

As a first effect of Covid, it should be noted that after the pandemic, slightly less than 50% of the rural tourism enterprises continued with their activity. Moreover, Covid has a significant influence on gender, age, educational level, being associated, and specific training received.

Additionlly, the results of our research show that there are relevant differences in promoters’ behaviour based on their gender. More specifically, there is a significant relationship between men’s participation in rural tourism associations and receiving public funds. By contrast, this connection was not significant in female promoters, so their associative tendencies are not statistically related to economic reasons. This result was found for both pre and post Covid periods.

Regarding the fulfilment of the hypotheses, after Covid, we observe that the presence of women rural tourism promoters increases, so hypothesis 1 is fulfilled. Specific training of the promoter increases significantly, but only for women. Finally, the promoter receives more subsidies as an associate, a result that is confirmed only for men. Hypotheses 2, 3, and 5 are therefore only partially fulfilled.

Hypothesis 4, which refers to the fact that being a member of an association guarantees the promoter greater access to specific training is confirmed for both men and women. Moreover, obtaining specific training increases the chances of promoters obtaining a subsidy, so hypothesis 6 is also confirmed for both men and women.

Regarding hypotheses 7 and 8, the results show that, on the one hand, the Covid effect significantly influences the advantage: "Facilitates access to foreign markets" and the disadvantage "It is a bureaucratic and unwieldy system". By gender, we found differences in one advantage "Simplifies the management of booking" which was significant for women, and in two disadvantages ("It is a bureaucratic and unwieldy system" and "Restricts individual freedom (prices, promotion, etc.)" significant for men.

On the other hand, we found that there are significant differences in the average ratings of all the advantages and disadvantages of associationism by both associates and non-associates. Consequently, hypothesis 9 is rejected.

Finally, hypotheses 10 and 11 are not fulfilled for the following items "The Covid situation has had a favourable influence on my interest in associationism". Therefore, the Covid influence is perceived differently by men and women. Moreover, "Individual (non-associated) management of rural accommodation is more difficult because of the Covid crisis", which is perceived differently depending on whether the promoter is associated or not. In all other cases, the impact of Covid is considered the same. In addition, similar results were obtained for pre and post Covid periods in some other items. For example, there is a link between receiving training in the sector and belonging to a rural tourism association in both periods.

To conclude, we focus on the area of recommendations.

On one hand, with respect to the differences by gender, men's interest in becoming members of an association has been influenced by Covid to a greater extent than for women. Therefore, bearing in mind the important role that networking can play in the development of rural areas, gender perspectives should be considered as well.

From another perspective, a significant positive relationship has also been found between obtaining public subsidies and belonging to a rural tourism association in both periods. Thus, networking clearly favours promoters’ access to these institutional funds to initiate ventures in the sector, especially in the early years of rural tourism on the island of La Palma, since subsidies were granted to real estate owners to renovate establishments for touristic purposes, which logically led to higher rates of networking. From that point onwards, rural tourism promotion policies in La Palma were focused on individualized support for natural persons’ business projects or commercial entities so that, once the first stage of creating rural tourism was over, future entrepreneurs in the field could decide whether or not to associate based on the pros and cons they perceived. Therefore, regarding public authorities, in broad terms, they should provide key elements for the development of rural tourism through an integral strategy aimed at creating synergetic activities in an area through subsidies for the refurbishment of old or deteriorated accommodation for touristic purposes, providing training, commercialization efforts, among others.

Finally, in the area of management recommendations, it is worth noting the value of training, before and after the Covid period, and the view rural tourism promoters interviewed in the post Covid period have on rural tourism as an option for the future. In this aspect, it is thought that the sector requires new differentiation strategies based on the values of the rural world and nature.

Specifically, the circumstances that have arisen for tourism as a result of the Covid-19 pandemic have led to the need for accelerated and profound changes in the preparation and provision of management services in accommodation. These changes were developed fundamentally in the confinement stage of the pandemic and have resulted in the adoption of compulsory health and hygiene protocols involving cleaning and disinfection systems, guest reception practices, protocols to be applied in the event of the appearance of positive Covid-19 cases, among others. Given the gender segregation of activities in accommodation management of rural tourism, in terms of dealing with guests, accommodation preparation (cleaning and disinfection), etc., special attention must be paid to training women in these protocols.

Furthermore, in a pandemic situation, accommodation establishments have had to deal with new procedures concerning, for example, recruitment, new rules on health passports or conditions to gain access to destinations and accommodation, established according to the requirements of each country. The above circumstances have resulted in the need for specialised skills among the people responsible for the management of rural tourism establishments. Fortunately, associations of rural tourism entrepreneurs are able to help in the application of these new protocols, and in their promotion, which will be very beneficial for the future of this activity.

In addition, the characteristics of rural tourism activities, in terms of their small business size and fragmented supply, have meant that public sector support, especially in the circumstances arising from the pandemic, has been channelled through associations. Associations can thus be seen as having an adjuvant role for the public administration. This role should be strengthened to the extent that these networks perform key functions such as providing professional information and guidance, familiarisation with complex bureaucratic procedures or facilitating access to processes and telematic delivery channels. It is perhaps these operational needs that have motivated men to associate to a greater extent than women in the aftermath of the pandemic.

### Electronic supplementary material

Below is the link to the electronic supplementary material.

Supplementary file1 (PDF 68 KB)

## Appendix 1: Questionnaire

Rate on a scale of 1–5, being 1 “not agree at all” and 5 “strongly agree” with the following statements:

**Table:**

Statements about Covid’s impact					
1.- Rural tourists will be more sensitive to health aspects after the Covid crisis	1	2	3	4	5
2.- Rural tourists will be more sensitive to environmental and climate change issues after the Covid	1	2	3	4	5
3.- Individual (non-associated) management of rural accommodation is more difficult because of the Covid crisis	1	2	3	4	5
4.- Rural tourism has been less affected than the rest of tourism by the Covid crisis	1	2	3	4	5
5.- The Covid situation has had a favourable influence on my interest in associationism	1	2	3	4	5
6.- Rural tourism could be an option for the future after Covid due to the interest of tourists in seeking isolation, nature and less overcrowding	1	2	3	4	5
7.- Booking platforms (Booking, Airbnb, etc.) will have more weight in generating bookings in rural tourism after Covid	1	2	3	4	5
8.- The Covid crisis has forced me to lower prices of my rural accommodation	1	2	3	4	5
9.- Direct public subsidies to rural accommodation are essential to cope with the loss of income caused by Covid	1	2	3	4	5
10.- A specific promotion of the values of rural tourism is necessary to differentiate it following the Covid crisis	1	2	3	4	5

**Table:**

Statements about rural tourism associationism					
1.- It unifies the image of the product	1	2	3	4	5
2.- It facilitates joint promotion	1	2	3	4	5
3.- It helps improve the quality of the offer	1	2	3	4	5
4.- It facilitates access to foreign markets	1	2	3	4	5
5.- It simplifies the management of bookings	1	2	3	4	5
6.- It is a bureaucratised and unwieldy system	1	2	3	4	5
7.- It restricts individual freedom -prices, promotion, etc	1	2	3	4	5
