# Supplementary material for: Rural tourism networking and covid-19 crisis: a gender perspective
Source: Serv Bus. 2022 Aug 27;16(4):1111–37. doi: 10.1007/s11628-022-00503-x (PMC9419131; doi:10.1007/s11628-022-00503-x)
Supplement: Supplementary file 1 — Supplementary file1 (PDF 68 KB) [file 11628_2022_503_MOESM1_ESM.pdf]

DATA SET:

https://doi.org/10.6084/m9.figshare.16892680.v4

https://doi.org/10.6084/m9.figshare.16892659.v4
